# Should I Trust the Artificial Intelligence to Recruit? Recruiters’ Perceptions and Behavior When Faced With Algorithm-Based Recommendation Systems During Resume Screening

**DOI:** 10.3389/fpsyg.2022.895997

**Published:** 2022-07-06

**Authors:** Alain Lacroux, Christelle Martin-Lacroux

**Affiliations:** ^1^Univ. Polytechnique Hauts de France, IDH, CRISS, Valenciennes, France; ^2^Univ. Grenoble Alpes, Grenoble INP, CERAG, Grenoble, France

**Keywords:** artificial intelligence, resume screening, trust, algorithmic decision support systems, algorithm aversion

## Abstract

Resume screening assisted by decision support systems that incorporate artificial intelligence is currently undergoing a strong development in many organizations, raising technical, managerial, legal, and ethical issues. The purpose of the present paper is to better understand the reactions of recruiters when they are offered algorithm-based recommendations during resume screening. Two polarized attitudes have been identified in the literature on users’ reactions to algorithm-based recommendations: algorithm aversion, which reflects a general distrust and preference for human recommendations; and automation bias, which corresponds to an overconfidence in the decisions or recommendations made by algorithmic decision support systems (ADSS). Drawing on results obtained in the field of automated decision support areas, we make the general hypothesis that recruiters trust human experts more than ADSS, because they distrust algorithms for subjective decisions such as recruitment. An experiment on resume screening was conducted on a sample of professionals (*N* = 694) involved in the screening of job applications. They were asked to study a job offer, then evaluate two fictitious resumes in a 2 × 2 factorial design with manipulation of the type of recommendation (no recommendation/algorithmic recommendation/human expert recommendation) and of the consistency of the recommendations (consistent vs. inconsistent recommendation). Our results support the general hypothesis of preference for human recommendations: recruiters exhibit a higher level of trust toward human expert recommendations compared with algorithmic recommendations. However, we also found that recommendation’s consistence has a differential and unexpected impact on decisions: in the presence of an inconsistent algorithmic recommendation, recruiters favored the unsuitable over the suitable resume. Our results also show that specific personality traits (extraversion, neuroticism, and self-confidence) are associated with a differential use of algorithmic recommendations. Implications for research and HR policies are finally discussed.

## Introduction

Human resources (HR) managers currently make extensive use of artificial intelligence– (AI–) based tools: a 2018 survey of 9,000 recruiters shows that 64 percent “use data at least “sometimes” in the course of their recruitment activity; 79 percent are likely to do so in the next 2 years, and 76 percent believe artificial intelligence will have a significant impact on recruiting” (LinkedIn, 2018). Indeed, International Data Corporation (2020) identifies AI-based HR tools as one of the fastest growth areas for corporate AI spending.

Typically, AI refers to the use of digital technology to create systems capable of autonomously performing tasks commonly believed to require human intelligence (Office for AI, 2019). As Burton et al. (2020) note, algorithm-assisted decisions cover a vast domain that includes related paradigms such as augmented decision making, decision aids, decision support systems, expert systems, decision formulas, computerized aids, and diagnostic aids. Herein, we use the term “algorithmic decision-support systems” (ADSSs) to characterize such systems, in which “outputs can be used as additional or alternative source[s] of information for decision makers” (Langer et al., 2021b, p. 753). These systems can serve multiple functions during the sourcing, pre-selection, and selection phases of the recruitment process. For example, resume-screening software can learn existing employees’ experience, skills, and other qualities and apply this knowledge to new applicants to automatically rank and shortlist the best applicants; AI technology can be used to search public data sources such as media profiles to get to know a candidate better; recruiters can use chatbots to interact with candidates in real time by asking questions to assess whether they match with the job requirements and provide feedback and suggestions; interviewers can use AI to assess the richness of candidates’ vocabulary, speech rate and tone, and facial expressions to assess fit for the job; and recruitment software integrating algorithms can match people according to personality traits and predict sustainable working relationships. Developers gather all these functionalities under the umbrella term “predictive hiring.”

A body of research investigates why and how people use ADSSs (Burton et al., 2020) and discusses whether people are averse to using recommendations generated by automated systems (Dietvorst et al., 2015) or if they appreciate and rely on such recommendations (Logg et al., 2019). In the domain of personnel selection, however, scant research addresses how ADSSs affect recruiters. The scientist–practitioner gap is large in terms of investigating how recruiters perceive different tools and approaches and why they do so (Kuncel et al., 2013; Chamorro-Premuzic et al., 2016; Gonzalez et al., 2019; Langer et al., 2019a).

Research demonstrates that human users (e.g., recruiters) may react in different ways to automation and suggests trust in decision quality and reliability as a major determinant for effective adoption of automation technology (Lee and See, 2004). In this context, we define trust as “the willingness of a party (the trustor) to be vulnerable to the actions of another party (the trustee) based on the expectation that the other will perform a particular action important to the trustor, irrespective of the ability to monitor or control that other party” (Mayer et al., 1995, p. 712).

**Figure 1:**
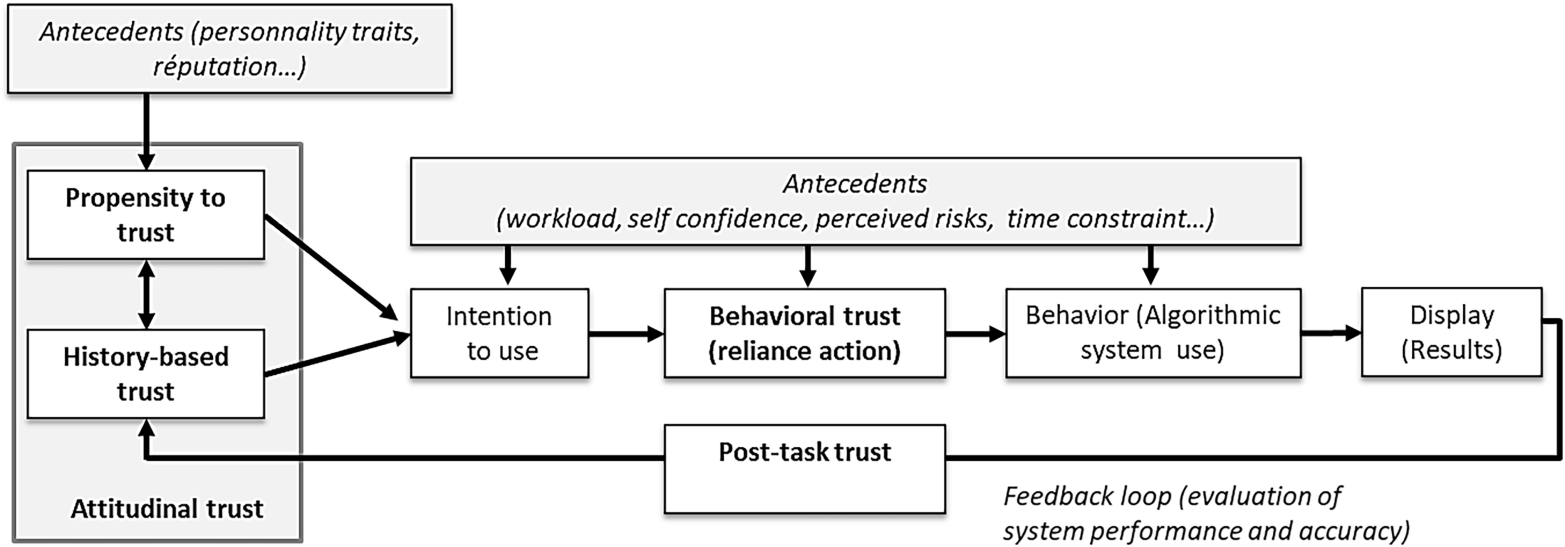
Dynamic of trust in human–ADSS interaction.

Researchers study trust in automated systems in the interpersonal context using two approaches: trust as either a stable disposition or a dynamic attitude that includes a behavioral dimension. Regarding trust as a stable disposition, Merritt and Ilgen (2008) suggested that individuals have a general propensity to trust or distrust a machine, just as they have a general propensity to trust or distrust another person. By contrast, other researchers viewed trust in automation as dynamic, such that it may vary depending on past experiences with the system, and that for trust to grow, people must first rely on automation (Muir and Moray, 1996). Building on previous work and their own findings, Merritt and Ilgen (2008) proposed a distinction between propensity to trust as a stable disposition (i.e., “In general, I trust ADSS”) and history-based trust based on past interactions with the automation (i.e., “From my own experience, I trust this ADSS”). Recent models of trust in ADSSs combined the stable and dynamic vision of trust in an integrative view. For example, Lee and See’s (2004, p. 68) conceptual model of dynamics governing trust involves a feedback loop in which the system’s behavior has a feedback effect on trust. In this model, attitudinal trust is an antecedent of reliance action, which can be considered the behavioral outcome of trust (i.e., “I trust the advice given by this specific ADSS, and I use it to inform my decision”). Over time, trustors evaluate recommendations made by the systems (or instantly, by contrasting with their own evaluation). This Post-Task trust that stems from experiencing the ADSS can lead trustors to revise their level of initial trust According to Merritt and Ilgen (2008), Post-Task trust contributes to building history-based trust (Figure 1).

Trust in automated systems plays a central role in Parasuraman and Riley’s (1997) typology of user reaction. These authors categorize user reactions into use, misuse, disuse, and abuse. “Use” refers to users who rely on automation to undertake tasks that they could perform manually: in such situations, a reasonable degree of trust in an automated system is necessary to engage in its use. “Misuse” refers to situations in which users overtrust the automation, even if it fails or behaves unpredictably. Also called overreliance on automation or automation bias, this overtrust signals that users are investing more trust in the automation than it merits (Parasuraman and Riley, 1997). “Disuse” refers to failures occurring when people reject the capabilities of automation (Lee and See, 2004, p. 50) because they do not trust the systems. Algorithm aversion occurs when users undertrust automated systems and do not rely on algorithmic decision or recommendation, preferring to rely on their own judgment (Dietvorst et al., 2015). “Abuse” is the automation of functions without regard for the consequences for humans or organizations (e.g., robotization leading to the replacement of human workers by machines). According to Parasuraman and Riley (1997), these varying reactions reflect a complex interaction of many factors, such as users’ feelings and attitudes (e.g., propensity to trust), risk, workload, and self-confidence. Among these factors, trust plays a crucial role; for example, overtrust leads to misuse and abuse, whereas the absence of trust leads to disuse. Thus, gaining a better understanding of the reasons managers use, misuse, or disuse certain selection procedures or tools is critical.

Glikson and Woolley’s (2020) review presents evidence of low initial trust in ADSSs, especially in field studies (in, e.g., health care, energy use), where mistrust can be so great that users refuse to use the embedded AI at all. However, the authors also note the scarcity of field studies that assess trust in ADSSs (which they refer to as “embedded AI”) in organizational settings. As mentioned previously, this scarcity is of particular concern for personnel selection, in which the market for predictive hiring tools is growing fast but academic research remains at an early stage (Campion et al., 2016; Langer et al., 2019b, 2021a; Tambe et al., 2019; Oberst et al., 2020). In personnel selection, the stakes are high because it involves ethic, legal, psychological, and strategic issues in organizations. Moreover, human intervention remains important: the recruitment process is not fully automated, which leaves room for the user to choose whether to use ADSSs. As Langer et al. (2021a, p. 3) note, this latitude for choice means that managers must assess the relative trustworthiness of humans and automated systems to decide whether to rely on ADSSs for a given task. A better understanding of the mechanisms governing this trust and its consequences on decision process for recruiters remains an important issue. However, translation of previous research on automation to the field of personnel selection is not straightforward and has not yet been fully explored. The issue of algorithmic overreliance or aversion in the context of personnel selection deserves greater attention.

Thus, in this research, we explore the relative impact of ADSS versus human recommendations and their consistence, as well as individual characteristics (e.g., propensity to trust, personality traits), on trust and subsequent behavior of professionals involved in preselection tasks (e.g., resume screening). In doing so, we aim to address Glikson and Woolley’s (2020) call for research considering the role of trust in AI within organizations. To this end, we conduct an online experiment, in which professionals in charge of personnel selection in their organization assess two applications in response to a job description with recommendations provided by either an ADSS or a human expert. We then examine the influence of the recommendation on participants’ perceptions and hiring decision.

## Trust and Distrust in Personnel Selection Context

### Trust and Source of Recommendations

Previous research established that the characteristics of the task being performed by ADSSs play an important role. When tasks involve human skills, users put more trust in a human expert than in an ADSS, such as when tasks involve subjective evaluation of work (Lee, 2018), require ethical and moral judgment (Bigman and Gray, 2018), or more generally have an impact on an individual’s fate (Longoni et al., 2019). All these characteristics apply to personnel selection, which makes trust in ADSS a critical issue for understanding their use in such a complex context. These characteristics also mean that completely delegating this task to machines is not realistic, and thus full automation of selection processes is not the goal; rather, predictive recruiting solution developers prefer to use the term “augmented recruitment” rather than “automated recruitment” (Raisch and Krakowski, 2021). Human intervention remains important, and thus studying trust in ADSS during personnel selection in conjunction with human expertise seems more relevant in a human–automation cooperation context.

During the recruitment process, applicant preselection (resume screening) is a crucial step for studying the impact of ADSSs for several reasons. First, automated systems already influence this stage to a great extent. Resume screening is a time-consuming task for which a wide range of solutions exists, from automatic extraction and applications’ ranking to advice on choosing the most suitable candidates, and ADSSs are already a viable option for decision support (Hickman et al., 2021). Second, resume screening is subject to heuristics, and research has demonstrated a significant impact of stereotypes and prejudices (Derous and Ryan, 2019): one of the strongest arguments in favor of ADSSs (other than time savings) is their “objectivity” and ability to reduce human biases. Third, resume screening is a crucial stage of the recruitment process, in which most candidates are eliminated, and thus can be considered risky with regard to ethical and legal aspects, especially when recruiters rely heavily on ADSSs.

To date, studies on trust in ADSSs during recruitment produced few results (Burton et al., 2020). Most studies revealed a tendency to disuse automation and highlight an aversion behavior. For example, Langer et al. (2021a) concluded that participants in a personnel selection context perceived the human trustee as more trustworthy than an automated trustee. The authors explained these results by noting that human ability is necessary to complete tasks that involved ethical issues. Convergent results showed that when the task is perceived as subjective or required human skills, it is considered more accurate and trustworthy when performed by a human rather than by an ADSS (Lee, 2018; Castelo et al., 2019). This preference for human recommendations emerges even if the recommendation is strongly subjective (Oberst et al., 2020). As a consequence, we argue that in the resume-screening stage, recruiters prefer human recommendation and are more influenced by this recommendation even if it is inconsistent (i.e., when the recommendation puts forward a less suitable candidate rather than a suitable candidate). Therefore, we posit the following:

*H1a*: Recruiters consider recommendations from a human expert more trustworthy than ADSS recommendations.

*H1b*: Recruiters are more influenced in their preselection task by human recommendations than ADSS recommendations.

In their review, Glikson and Woolley (2020) observed a stable pattern, indicating that errors and inconsistencies of embedded AI are detrimental to cognitive trust. When ADSSs make errors, users’ trust in and reliance on the ADSSs significantly decrease (Dzindolet et al., 2003). In line with these results, Dietvorst et al. (2015) highlighted the detrimental effect of an error: users prefer to rely on human rather than algorithmic forecasts, even in case of human errors. Therefore, we posit the following:

*H2a*: Recruiters consider an inconsistent recommendation from a human expert more trustworthy than an inconsistent ADSS recommendation.

*H2b*: Recruiters are more influenced in their preselection task by an inconsistent human recommendation than an inconsistent ADSS recommendation.

### Trust and Personal Characteristics of the User

Beyond the characteristics and performance of ADSSs, individual users’ personality and disposition are recognized as important determinants of trust in automation (Schaefer et al., 2016). For example, individuals with greater domain expertise are less likely to trust automation than novice operators. Sanchez et al. (2014) found that individuals with agricultural driving experience are more reluctant to rely on automated alarms during a collision avoidance task than individuals with little or no experience in the agricultural domain. Similarly, in an experiment manipulating industry familiarity, participants who possess industry expertise rely less on an AI assistant when it provides erroneous outputs (Dikmen and Burns, 2022). The same trend was identified by Lee and Moray (1994) and Waern and Ramberg (1996) regarding subjects’ self-confidence in their ability to perform a task unassisted. Other studies showed that dispositional traits (e.g., general propensity to trust, self-confidence) affect trust in ADSS recommendations. Propensity to trust automated systems appeared as an antecedent of trust behavior (reliance action), in line with the dynamic model of trust in automation (Singh et al., 1993; Lee and See, 2004; Jessup et al., 2019). The majority of these results comes from studies conducted on tasks involving human–machine collaboration. We acknowledge that the tasks described in these studies were often less complex than those performed by recruiters; however, considering that personality and dispositional traits are relatively independent of the context of the human–machine interaction (because they are generally considered antecedents of human–machine interaction behaviors), we propose a transposition of the previous results to the use of ADSS in a resume-screening task and therefore posit the following:

*H3a*: Recruiters’ propensity to trust in ADSSs is positively associated with trust in ADSS recommendations.

*H3b*: Self-confidence in recruitment (i) is negatively related to propensity to trust algorithms and (ii) leads to lower trust in ADSS recommendations.

*H3c*: Expertise in recruitment (i) is negatively related to propensity to trust algorithms and (ii) leads to lower trust in ADSS recommendations.

Research also highlighted associations between several personality traits and the propensity to trust automated systems (i.e., dispositional trust). In terms of the Big Five personality traits, trust has been primarily analyzed in situations involving interpersonal interactions, and some results were extended in situations involving human–automation interactions. In an interpersonal context (expert advice), studies concluded that trust is positively related to extraversion and negatively related with neuroticism; trustworthiness is shown to be positively related to agreeableness and conscientiousness (Evans and Revelle, 2008). In a recent study, a negative association was found between propensity to trust other people and neuroticism, and a positive correlation was found for the four other Big Five traits (Zhang, 2021). Research on trust in human-machine interaction led to scarce and unconclusive results: in some studies, neuroticism is the only trait that correlates (negatively) with agreement with automated systems (Szalma and Taylor, 2011; McBride et al., 2012). Extraversion has been found to be associated to a greater propensity to trust machines (Merritt and Ilgen, 2008). Conversely, in a multicultural research, authors found a positive association between propensity to trust automation and two big five traits not mentioned in previous studies: conscientiousness and agreeableness (Chien et al., 2016). Therefore posit the following:

*H3d*: Extraversion is positively related to propensity to trust algorithms.

*H3e*: Neuroticism is negatively related to propensity to trust algorithms.

*H3f*: Agreeableness is positively related to propensity to trust algorithms.

*H3g*: Conscientiousness is positively related to propensity to trust algorithms.

## Materials and Methods

### Development of Study Materials

First, we developed a job description for an HR Manager position inspired from similar positions posted on job sites. Second, we developed two resume abstracts using the same template and sections based on actual resumes posted on job sites (name, home and email address, educational degree, work experiences, competency statement, and foreign language proficiency). We differentiated the two abstracts by manipulating the level of work experience, educational attainment, foreign language proficiency, and managerial skills, such that one of the resumes was exactly in line with the job description and the other was significantly less suitable. To enhance realism, we made both resume abstracts consistent with the job offer. Four HR management experts reviewed the material to ensure realism and suitability.

In the third stage, we created five conditions regarding the recommendations accompanying the resumes. The human expert’s recommendations consisted of excerpts from phone conversations with a recruitment agency head (the human expert) that the recruiting company had used several times for former recruitments.

Participants learned that the recruitment agency head had received the job description and the two resumes before making a recommendation. The algorithmic recommendations consisted of a predictive recruitment solution developed by a fictional startup (HR Predict) that specialized in the development of advanced algorithmic solutions, integrating the latest developments in AI (machine learning and deep learning). Participants were exposed to recommendations from HR Predict, which proposed to rank applicants by their matching rate to the job description.

The five conditions regarding the recommendations were the following: no recommendation, a consistent human recommendation, an inconsistent human expert recommendation, a consistent algorithmic recommendation, an inconsistent algorithmic recommendation.

### Participants

In this research, we analyzed participants’ trustworthiness and subsequent behaviors (i.e., scoring and ranking application forms) after being exposed to recommendations. To enhance ecological validity of our results, we selected a sample of experienced professionals involved in the recruitment process, in contrast with previous research on trust in selection context, in which participants involved were mainly students or working adults (Diab et al., 2011; Dietvorst et al., 2015; Dietvorst, 2016; Lee, 2018; Langer et al., 2021a) and only a few studies used participants actually involved in recruitment tasks in their organization (Oberst et al., 2020). We selected participants from an online panel owned by a specialized company, Panelabs, which gives access to a panel that contains 500,000 French participants and provides a high level of quality control. Marketing and organizational researchers are increasing using online panel data to obtain convenience samples (e.g., Bowling and Lyons, 2015; Djurdjevic et al., 2019). We obtained 694 usable responses over a two-week period (134 in a first collection and 560 in a second collection). Respondents’ age ranged from 20 to 69 years, with a mean of 40.5 years (SD = 9.97); 47.9% were male and 52.1% female. They were employed in companies ranging in size from 10 to more than 1,000 employees (with a median class of 50–199). Regarding their professional experience in personnel selection, 50.8% of the respondents had three or more years’ experience, and 34.4% had one to 3 years’ experience.

### Procedure

**Figure 2:**
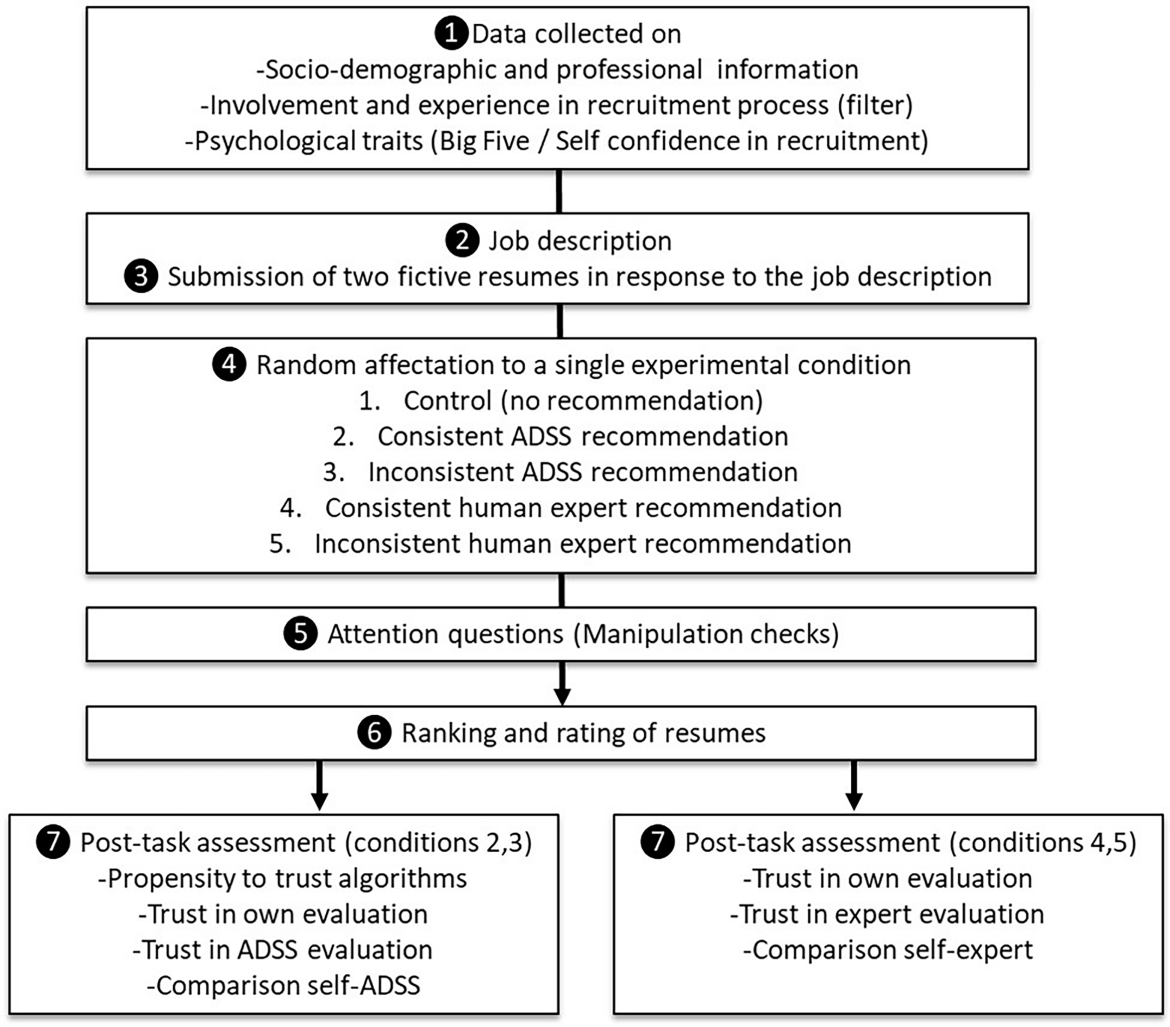
Overview of experimental procedure.

Participants voluntarily completed the experiment online. The experimental procedure is summarized in Figure 2.

First, participants had to answer a series of questions (1). Then, they read a job description for an HR Manager (2) followed by two resume abstracts (3) in which the applicants were unequally qualified (see “Development of Study Materials”). We counterbalanced the applicants’ resumes to avoid order effects. Participants received synchronous recommendations: they were randomly exposed to five conditions (4) in a 2 × 2 between-subjects design, with a control group in which participants were not exposed to recommendations. Manipulated factors were the type of recommendation (human vs. algorithmic) and the relevance of recommendation (consistent vs. inconsistent).

As previously stated, we considered a recommendation for the least suitable application as inconsistent and that for the most suitable candidate as consistent. As part of this filler task, we also asked participants to complete items to check their attention during the task and identify the differences between the two resumes (5). Participants who successfully answered attention questions were finally asked to report their levels of trust in recommendations provided by either the ADSS or the human expert. They were asked to choose the best application and to rate both of them by responding on a 10-point scale (6).

### Measures

Sociodemographic information (age and gender) was collected to be used as control variables. For these variables, authors generally conclude that the context plays a prominent role. According to Hoff and Bashir (2015), *Age* can influence trust in automation, as older adults tend to comply with and rely on automation more than younger adults. Pak et al. (2017) investigated trust in technology across different groups (young adults, military and older adults) and domains and found that trust in automation may vary in distinct context. Relatively few studies examined *gender differences* in relation to trust in automation and of those that have, findings are inconsistent (Hoff and Bashir, 2015). No gender differences are highlighted regarding trust in automation, but males and females may respond differently to an ADSS (Lee, 2018).

We measured participants’ *professional expertise in recruitment* with two questions: “How many years have you been involved in these selection/recruitment procedures?” and “On average, during each of these selection procedures, how many applications do you screen?” We computed a composite score representing expertise in recruitment as log(experience × number of applications screened). We also asked participants if they had already *use an ADSS* in their professional practice recoded in a binary item (yes/no).

We assessed *participants’ personality* using a 10-item short version of the Big Five inventory (Rammstedt and John, 2007); a sample item is: “I see myself as someone who is relaxed, handles stress well” (mean Cronbach’s *α* = 0.57).

To measure participants’ *self-confidence in their ability to recruit*, we adapted Williams and Deci’s (1996) perceived competence scale (4 items); a sample item is: “I feel confident in my ability to conduct a successful recruitment” (Cronbach’s *α* = 0.91). To assess their perceived confidence with the task, we also measured participants’ *trust in their own decisions* using items proposed by Dietvorst (2016), scored on a 9-point Likert scale (e.g., “How confident are you in your own decision?”).

To assess *the influence of recommendations on participants’ behavior*, we used two types of measures. First, we computed the difference of scores between the suitable and unsuitable resumes (diff-score variable): the larger this difference (in comparison with the control group), the more influential the recommendation was. A negative diff-score for a particular condition indicates that participants preferred the unsuitable to the suitable resume. Second, we computed and compared the frequencies of resumes that were ranked in first place.

We measured *trust in the human expert or ADSS recommendations (Post-Task trust)* with two items proposed by Dietvorst (2016) and scored them on a 9-point Likert scale: “How trustworthy do you consider the recommendation provided by [the expert/the algorithmic solution]?” These measures allow us to assess Post-Task trust in the expert or automation, given that we took them after task completion. We have added two items asking participants to *compare their decisions to ADSS or expert recommendation* with two items proposed by Dietvorst (2016) and scored on a 9-point Likert scale: “How trustworthy do you consider the recommendation provided by [the expert/the algorithmic solution]?” We assessed *propensity to trust automated systems (dispositional trust)* for participants randomly exposed to the algorithmic conditions using the trust between people and automation scale adapted from Jian et al. (2000); two sample 7-point Likert items are: “ADSS are deceptive” (reverse-coded) and “ADSS are reliable” (Cronbach’s *α* = 0.84). This scale comprises nine items.

### Pilot Study

We conducted a pilot study on the initial sample of 134 recruiters supplied by the online survey company. Our goal was to check that the two resumes were correctly distinguished and that the manipulations of recommendations were effective. The results showed that in the control group (no recommendation condition), participants rated the two applications as significantly differentiated (mean difference = 0.26; paired *T*-test: *t*(163) = 3.32, *p* = 0.018) and the suitable resume was more often ranked first than the unsuitable one (respectively, 62.5 and 37.5%). Regarding the impact of experimental conditions, an analysis of variance (ANOVA) of the difference between scores for the two resumes revealed that this impact significantly differed by experimental conditions (*F*(4, 159) = 2.47, *p* = 0.04, eta^2^ = 0.06). These results suggest that the experimental material was understandable: participants took into account the recommendations when assessing the two applications. Data from the pilot study were then pooled with data from the second data collection (*N* = 530) to be used in subsequent statistical procedures.

### Analytical Strategy and Results

Correlation matrix with descriptive statistics of variables used in the study is presented in Supplementary Table S1. Descriptive statistics and distribution plots of scores for dependent variables (violin plots) are presented (Supplementary Figures S1–S4).

We used a regression-based framework with contrast analysis (pairwise comparisons on estimated marginal means) to test hypothesis H1 and H2 related to experimental manipulations. We first estimated simple models with experimental conditions as dummy variables (stage 1), then we estimated models of trust including other independents and control variables (stage 2) in order to test H3 hypotheses related to trust behavior.

Five models were estimated: three models with dependent variables diff-scores and factors modeling experimental conditions (Table 1), followed by two models with Post-Task Trust as dependent variable (Table 2) including control variables (personality traits and demographic variables). We estimated two models, distinguishing Post-Task Trust in human expert recommendation and Post-Task Trust in ADSS recommendation. Calculations were made in R statistical environment, using JASP software (JASP Team, 2022), along with R packages ggstasplot (Patil, 2021) and emmmeans (Lenth, 2018). Raw data, R scripts, and vignettes used in the experiment are available in https://osf.io/t5wsq/.

**Table 1:** Impact of experimental conditions on trust and behavior (regression models).

	Trust in recommendation	Dependant variable	
		Diff_score (1)	Diff_score (2)
	Model 1 (OLS)	Model 2 (OLS)	Model 3 (OLS)
ADSS recommendation		−0.34^*^(0.145)	
Expert recommendation	0.48^**^ (0.145)	0.001 (0.146)	0.14 (0.166)
Inconsistent recommendation	−0.13 (0.142)		−0.83^***^(0.163)
Expert rec. × Inconsistent rec.	−0.08 (0.203)		0.38 (0.234)
Constant	6.63^***^ (0.102)	0.54^***^ (0.119)	0.63^***^ (0.117)
Observations	557	694	557
*R* ^2^	0.038	0.014	0.070
*F* Statistic	7.24^***^ (*df* = 3; 553)	5.05^***^ (*df* = 2; 691)	13.86^***^ (*df* = 3; 553)

SE in parentheses; Models 1 and 3 do not include control group; reference groups = control group (model 2); ADSS/consistent recommendation group (models 1 and 3).

* *p* < 0.05;

** *p* < 0.01;

*** *p* < 0.001.

**Table 2:** Trust perceptions according to the source of recommendation.

	Dependent variable	
	Post-Task trust in expert recommendation	Post-Task trust in ADSS recommendation
	Model 4 (OLS)	Model 5 (OLS)
Recommendation (inconsistent)	−0.215 (0.124)	−0.126 (0.123)
Recruiter expertise	−0.009 (0.085)	0.053 (0.085)
Self-competence in recruitment	0.214^***^ (0.062)	0.189^**^ (0.068)
ADSS user (yes)	0.023 (0.129)	−0.269^*^ (0.131)
Age	−0.005 (0.007)	−0.016^*^ (0.007)
Gender (female)	−0.094 (0.130)	0.146 (0.124)
Propensity to trust automation		0.756^***^ (0.060)
Extraversion	0.025 (0.050)	0.059 (0.050)
Agreeableness	0.089 (0.061)	0.052 (0.062)
Conscientiousness	0.222^***^ (0.063)	0.008 (0.068)
Neuroticism	0.004 (0.052)	0.040 (0.053)
Openness	0.043 (0.049)	0.012 (0.053)
Constant	4.181^***^ (0.659)	1.938^**^ (0.675)
Observations	270	287
Adjusted *R*^2^	0.116	0.391
*F* statistic	4.213^***^ (*df* = 11; 258)	16.284^***^ (*df* = 12; 274)

* *p* < 0.05;

** *p* < 0.01;

*** *p* < 0.001.

With regard to the hypotheses on trust, H1a stated that recruiters would consider a human expert recommendation more trustworthy than an ADSS recommendation (Table 1, Model 1). Indeed, simple contrast analysis showed that the means of perceived trust by source of recommendation differed in support of H1a (Estimated difference: expert-ADSS = 0.437, *t*(553) = 4.29, *p* < 0.001). H2a stated that recruiters would consider inconsistent human expert recommendations more trustworthy than inconsistent algorithmic recommendation (Table 1, Model 2). Contrast analysis showed that the means of perceived trust by source of inconsistent recommendation type differed (Estimated difference: inconsistent expert-inconsistent ADSS = 0.396, *t*(553) = 2.77, *p* = 0.006), in support of H2a.

For the hypotheses investigating recruiters’ behavior, H1b posited that recruiters would be more influenced by human recommendation than by ADSS recommendation (i.e., compared with the control group, human recommendation increases the difference in scores between the suitable and unsuitable resumes more than algorithmic recommendation does). Regression model 2 showed a non-significant impact of expert recommendation compared to control group (*B* = 0.001, *p* = 0.99), though we observed a significant difference between the ADSS recommendation condition and the control group (*B* = −0.34, *p* = 0.02). Therefore, H1b was not supported. Our results suggested that recruiters were more influenced by algorithmic recommendation than by human expert recommendation and that this influence was not in the expected direction (the difference in scores between suitable and unsuitable resumes decreased). The analysis of ranking decisions confirmed this tendency: the percentage of suitable resumes ranked in first position when participants received human recommendation (67.03%) was higher than that in the control group (64.2%), but the difference was nonsignificant according to a Chi-squared test (Chi^2^ (407) = 0.32, *p* = 0.57, OR = 1.13). Conversely, the percentage of suitable resumes ranked in first position when participants received ADSS recommendation (56.1%) was lower than that in the control group (64.2%); the difference was greater than in previous case but still non-significant (Chi^2^ (424) = 2.53, *p* = 0.12, OR = 0.71). However, the difference in resume ranking in first position after expert or ADSS recommendation was statistically significant (respectively 67.03 and 56.1%; Chi^2^ (557) = 7.02, *p* = 0.008, OR = 1.59).

To further investigate this behavior, we proposed H2b, which posited that recruiters would be more influenced by inconsistent human recommendation than by inconsistent algorithmic recommendation. Regression model 3 showed that the consistence of the recommendation had a stronger impact (*B* = −0.83, *p* < 0.001) than its source (*B* = 0.14, *p* = 0.41). An inconsistent recommendation reduced the difference in score between resume, which means that recruiters were influenced by suggestion in favor of the least suitable resume.

**Figure 3:**
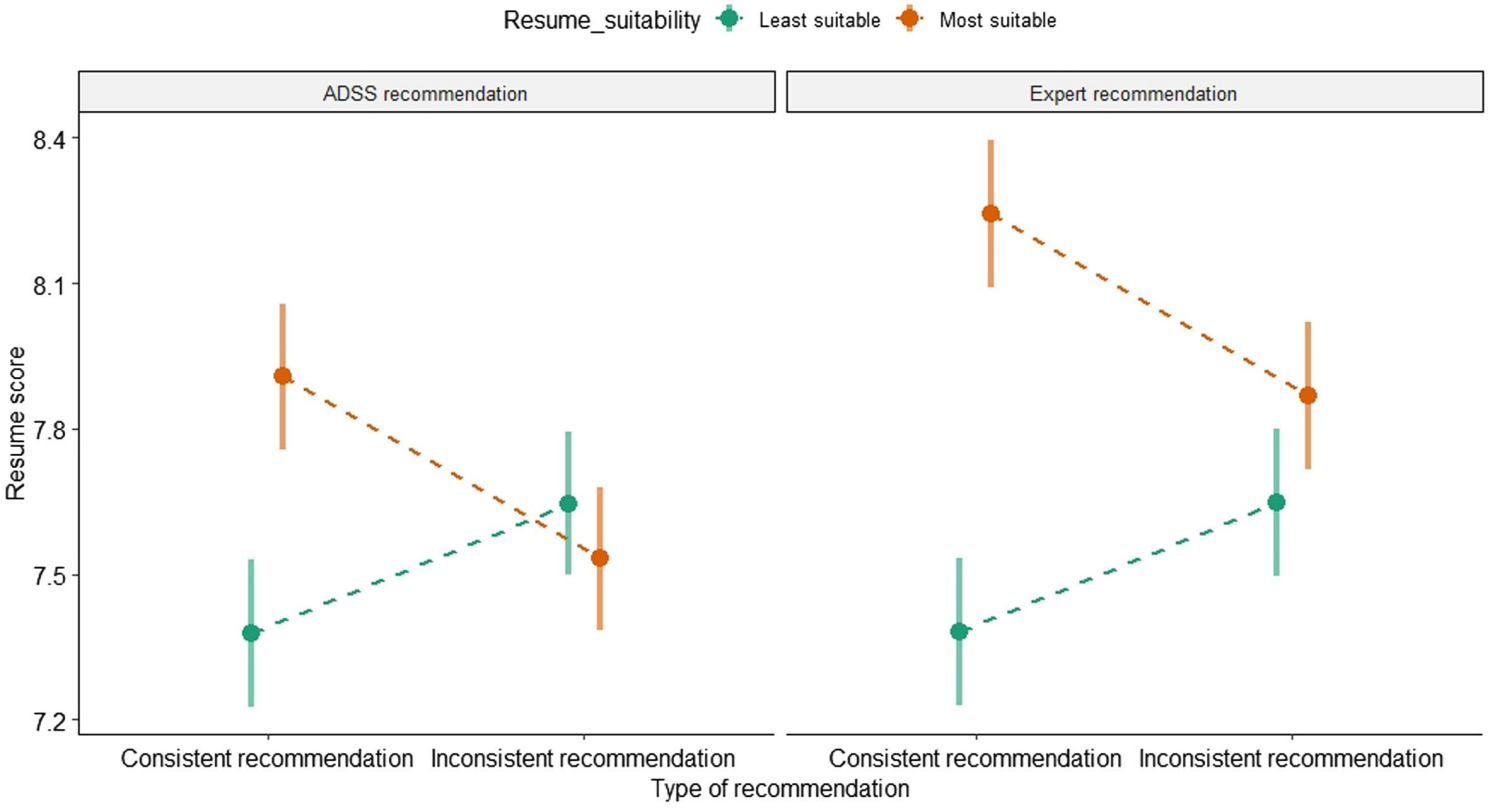
Influence of recommendations type and consistence on resume score.

Contrast analysis between experimental and control conditions (Table 3) revealed a significant difference between ADSS and human inconsistent recommendation, but in a different direction than expected. Compared to control group, participants were *not* influenced by inconsistent expert recommendation, but they were influenced by inconsistent ADSS recommendation. In the presence of an inconsistent algorithmic recommendation, recruiters favored the unsuitable over the suitable resume by giving a higher score, as shown in Figure 3. Therefore, the data did not support H2b.

**Table 3:** Contrast analysis (diff-scores depending on source and consistence of recommendations).

Contrasts	Estimate	SE	*df*	*t*-ratio	*p*-value^*^
ADSS consistent—control	0.086	0.164	689	0.522	0.985
ADSS inconsistent—control	−0.743	0.162	689	−4.593	0.000
Expert consistent—control	0.223	0.165	689	1.347	0.662
Expert inconsistent—control	−0.222	0.165	689	−1.340	0.666
ADSS inconsistent—ADSS consistent	−0.829	0.161	689	−5.142	0.000
Expert inconsistent—Expert consistent	−0.444	0.166	689	−2.677	0.058

* Tukey correction for multiple comparison.

Analysis of ranking decisions (Supplementary Figure S4) confirmed this tendency: the percentage of least suitable resumes ranked in first position was higher when participants received inconsistent algorithmic recommendation (55.4%) than when they received inconsistent human recommendation (40%), and the difference was significant according to a Chi-squared test (Chi^2^ (283) = 6.7, *p* = 0.01, OR = 1.86), meaning that participants were more influenced by inconsistent algorithmic recommendation, leading them to favor the unsuitable resume.

To identify the association of personality and dispositional traits with trust and behavior toward ADSS, we examined the correlation matrix (Supplementary Table S1) and regression models. Correlation matrix revealed weak correlations between propensity to trust automation and personality and dispositional traits, in majority not in line with hypotheses H3 (Table 4).

**Table 4:** Hypothesis H3 test (correlations/regressions).

Hypothesis	Result
H3a: Recruiters’ propensity to trust in ADSSs positively associated with Post-Task trust.	Supported
H3b: Self-confidence in recruitment	
(i) negatively related to propensity to trust automation and	Not supported
(ii) leads to lower Post-Task trust in ADSS	Not supported
H3c: Expertise in recruitment	
(i) negatively related to propensity to trust algorithms	Not supported
(ii) leads to lower Post Task trust in ADSS	Not supported
Propensity to trust algorithm	
H3d: Extraversion positively related to propensity to trust ADSS	Supported
H3e: Neuroticism negatively related to propensity to trust ADSS	Not supported
H3f: Agreeableness positively related to propensity to trust ADSS	Supported
H3g: Conscientiousness positively related to propensity to trust ADSS	Not supported

Regression models with control variables (Table 2, models 4 and 5) showed that propensity to trust ADSS strongly affect Post-Task trust for ADSS (*B* = 0.756, *p* < 0.001), and self-competence actually influenced Post-Task trust in ADSS and expert recommendation, but not in the direction that was expected in H3b. Recruiter’s expertise had no significant effect on Post-Task trust, while ADSS use in professional practice had a negative impact on Post-Task trust in ADSS. We also noticed that Big Five traits did not significantly influence Post-Task Trust in ADSS, while conscientiousness only significantly influenced Post-Task trust in human recommendation. This differential impact of personality traits on trust provided an additional argument in favor of a substantial difference in the nature in trust between human and ADSS experts, consistent with the findings of Madhavan and Wiegmann (2007).

Overall, our hypotheses H3 received mixed support. Personality traits and dispositions had a limited influence on attitudes toward algorithmic recommendations and subsequent behavior in our sample (Table 4).

To refine the analysis by taking into account the impact of recommendations’ consistence identified in H2b, we computed correlations between personality traits and Post-Task Trust on subsamples and distinguished between consistent and inconsistent conditions (Table 5). When recruiters were exposed to inconsistent algorithmic recommendations, extraversion influenced their trust behavior. This finding could be explained by the positive emotions facet of extraversion (Costa and McCrae, 1992), which may have increased the credibility to inconsistent or persuasive information as peripheral route of persuasion (Petty and Cacioppo, 1986). Subsequently, extraversion which is based on positive affect may have been positively associated with trust to automated systems. By contrast, our results suggested that when recruiters high in neuroticism were exposed to inconsistent algorithmic recommendation, they were significantly less influenced in their Post-Task trust perception, an unsurprising result considering individuals high in this trait tend to be more cautious and to make their decisions more carefully.

**Table 5:** Pairwise correlations between personality traits and Post-Task trust in ADSS recommendations (consistent vs. inconsistent conditions).

Consistent condition	Pearson *r*
Trust_in ADSS recommendation: extraversion	0.12
Trust_in ADSS recommendation: agreeableness	0.10
Trust_in ADSS recommendation: conscientiousness	0.09
Trust_in ADSS recommendation: neuroticism	0.03
**Inconsistent condition**	**Pearson *r***
Trust_in ADSS recommendation: extraversion	0.21^**^
Trust_in ADSS recommendation: agreeableness	0.11
Trust_in ADSS recommendation: conscientiousness	0.04
Trust_in ADSS recommendation: neuroticism	−0.19^*^

* *p* < 0.05;

** *p* < 0.01.

To conclude this section, we discuss the unexpected result observed in Figure 3: when recruiters were exposed to an inconsistent algorithmic recommendation, they considered that the recommendation was unreliable (see Supplementary Figure S2), but they were influenced in their choice. This behavior is typical of automation bias, when users blindly follow faulty algorithmic advice (Parasuraman and Manzey, 2010). We cannot conclude that the choice of the least suitable CV was a direct consequence of automation bias, as illustrated by the outcomes of the control group (the least suitable resume was chosen in 35.8% of the cases, without any recommendation). Alternative explanations could be proposed: for instance, the participants might have assumed that the ADSS or the human expert picked up on some cue that they did not pick up on. They might think that ADSS can see the hidden meaning of some piece of info in the application forms that they did not understood and then combine their own impression with the ADSS/expert recommendation.

Still, we chose to explore predictors of this paradoxical overtrust in inconsistent ADSS recommendation on the subsample in which participants were exposed to such a condition (*N* = 156). To this end, we created a binary variable corresponding to the situation in which the ADSS recommended the least suitable resume and the participants ranked it first. We then used logistic regression to identify possible predictors (Table 6). We entered the variables used previously (i.e., Big Five traits, the recruiter’s gender and age, the recruiter’s expertise, and the participants’ comparison of their own assessment to the ADSS) into the model. To further explore the results, we used dominance analysis applied to logistic regression (Azen and Traxel, 2009). This exploratory technique aims to evaluate the importance of the predictors in terms of their relative contribution to the pseudo-*R*^2^, taking into account that the predictors may be correlated. The objective was to isolate the overall contribution of each predictor in all possible configurations.

**Table 6:** Predictors of inconsistent ADSS’ recommendation influence (logistic regression).

	Dependent variable: choice of least suitable resume under inconsistent ADSS recommendation	
	Coeff (logit)	Odd ratio (CI 95%)
Recruiter expertise	0.361^*^ (0.187)	1.435 (1.069, 1.802)
Self-competence in recruitment	0.059 (0.159)	1.061 (0.749, 1.372)
Compare self with ADSS	0.322^**^ (0.122)	1.381 (1.142, 1.619)
Trust in own evaluation	−0.331 (0.174)	0.718 (0.377, 1.060)
Trust in ADSS recommendation	0.267 (0.163)	1.306 (0.987, 1.625)
Extraversion	−0.034 (0.112)	0.967 (0.748, 1.186)
Agreeableness	−0.043 (0.142)	0.958 (0.678, 1.237)
Neuroticism	0.028 (0.118)	1.028 (0.797, 1.259)
Conscientiousness	−0.324^*^ (0.146)	0.724 (0.437, 1.010)
Openness	−0.006 (0.116)	0.994 (0.767, 1.222)
Constant	−1.139 (1.621)	0.320 (−2.857, 3.497)
Observations 287	Pseudo *R*^2^ (Nagelkerke) 0.115	
Log likelihood −159.7	Akaike Inf. Crit. 341.4	

Predictors’ relative importance (dominance analysis): Compare self with ADSS (39%); Conscientiousness (19.1%); Trust in ADSS (17.4%); Recruiter expertise (14.8%); Trust in own evaluation (7.8%); other predictors contribution (< 1%).

* *p* < 0.05;

** *p* < 0.01.

Results (Table 6) showed that predictors had contrasted impacts. Conscientiousness and trust in own evaluation reduced the influence of the inconsistent ADSS’ recommendation but, surprisingly, the influence of ADSS appeared to be positively related to recruiters ‘expertise. The most important predictor of the inconsistent ADSS’ recommendation influence was the overconfidence of the recruiter in his/her superiority compared to ADSS, and the most important predictors of the reduction of the ADSS’ inconsistent recommendation influence were conscientiousness and trust in own evaluation.

## Discussion

### Overall Findings

Given the paucity of research on trust processes in domains in which ADSSs influence decisions that affect the fate of individuals, our results contribute to a better understanding of such trust processes. Our study explored the mechanisms underlying recruiters’ behavior toward ADSSs. The most noticeable result is paradoxical. On the one hand, recruiters trusted human expert recommendation more than the ADSS recommendation, consistent with previous research. This finding held even when the ADSS recommendation was inconsistent: recruiters considered the inconsistent human expert’s recommendation more trustworthy. In line with Langer et al. (2021a), our results indicated that people did not assume high performance for automated systems in personnel selection, as Post-Task trustworthiness assessments were lower for automated systems.

On the other hand, recruiters were more influenced by the ADSS in their behavior, when they had to rank the applications. Even when recommendations provided to recruiters were inconsistent, these recruiters were still more influenced by the algorithmic recommendation. This result implies that recruiters may have been convinced that ADSSs were less likely to make mistakes or believed that ADSSs were less biased than human experts. Therefore, our results highlighted a strong ADSS’ recommendation influence. These results are consistent with Dijkstra et al. (1998), who used a framework in which participants had to make judgment about a criminal law case (with or without expert system): in this framework, comparable with a recruitment decision in terms of subjectivity and complexity, participants often agreed with an incorrect advice of an expert system, leading them to a lower accuracy in their decision compared to a control group who did not receive any advice. Our findings are not completely in line with the widespread conclusion that people distrust algorithms in the domain of employee selection and hiring decisions. We found that participants reported greater trustworthiness of human experts (in line with Diab et al., 2011; Lee, 2018; Oberst et al., 2020), but they did not behave accordingly in the presence of inconsistent algorithmic recommendations.

Examination of predictors of trust in recommendation (models 4 and 5) highlights differences in the nature of trust between algorithm and human experts: age seemed to have an influence on trust in ADSS recommendation, but not in trust in expert recommendation: older recruiters were less likely to trust ADSS recommendation. This effect is not in line with Ho et al. (2005), but confirms conclusions from literature reviews that stress the importance of the taking into account context (e.g., type of task and experimental situation) for studying impact of socio demographic factors on automation use (Madhavan and Wiegmann, 2007; Schaefer et al., 2016; Pak et al., 2017).

Concerning personality traits, we found no significant impact on trust behavior, except for the influence of conscientiousness on trust toward human expert recommendation. Regarding conscientiousness, our findings were not consistent with other research suggesting that trust is negatively related to conscientiousness (Freitag and Bauer, 2016). People high in this personality trait are more cautious and better informed, make decisions carefully, and do not easily trust other people’s actions or decisions. Moreover, they generally consider themselves highly competent (McCrae and Costa, 2003), which could explain why they seemed to be less influenced by inconsistent algorithmic recommendations in our study.

Regarding the influence of the ADSS recommendation, our results suggest that recruiters with greater expertise are more likely to rely on an inconsistent algorithmic recommendation (*B* = 0.36, *p* = 0.053), in contrast with prior research indicating that individuals with greater subject matter expertise were less likely to rely on automation than operators with less expertise (Sanchez et al., 2014; Dikmen and Burns, 2022). To explain this paradoxical result, we note that the level of expertise declared by recruiters concerned a “traditional” recruitment process (not an algorithm-assisted recruitment process). Thus, their professional expertise was not easily transferable to ADSS: most ADSSs based on AI are opaque to their users, for technical (use of unsupervised learning algorithms) and commercial (industrial secret) reasons. This “black box problem” is an emerging issue in HR management. For example, attitudes of users confronted with predictable but opaque and inexplicable ADSSs seem to increase algorithmic aversion, according to an interview-based study (Ochmann et al., 2021). In our sample, only 5% of recruiters reported using ADSSs regularly, and 63.1% had never used one. Thus, we argue that recruiters may not have been aware of the black box problem, and this ignorance encouraged them to follow the recommendations, even though the ADSS recommendation was inconsistent. Examination of Model 5 (Table 4) supports our suggestion: ADSS users were less likely to trust ADSS recommendation (*B* = −0.26*, p* = 0.004). Again, these findings are in line with our results: lack of trust did not directly predict influence of ADSSs on recruiters’ decisions.

### Limitations and Further Research Opportunities

Certain limitations remain that should be addressed in future research. First, the task assigned in this study was complex and subjective, in comparison with those proposed in similar experimentations comparing differential impact of ADSS and human expert advices (see, for example Lee and Moray, 1992; Singh et al., 1993; Dzindolet et al., 2001). Recruiter’s choice reflected a part of subjectivity (this can be seen in the choices made by the control group in which the least suitable resume was selected by 35.7% of respondents). As a consequence, it remains difficult to isolate the unique influence of recommendation in recruiter’s choice. Accordingly, we found small effects of recommendations on trust and behavior (*R*^2^ for regression models range from 0.04 to 0.39); we also attribute these small effects to our concern about ecological validity, which led us to create credible application forms whose characteristics were consistent to the job offer, and these applications did not differ appreciably.

Some other variables identified in the literature could be taken into account to shed more light on the antecedents of recruiters’ trust behavior (e.g., workload, time constraints; Lee and See, 2004). We also recommend studying the effects of human and algorithmic recommendations provided at the same time to evaluate their respective influence and possible interactions.

An important issue to be addressed in future work on ADSSs is the context of use, in connection with the *black box problem. In the field of technology acceptance, Kroenung and Eckhardt (2015) propose an important distinction between voluntary and mandatory use of ADSSs. In our work, as in many situations in real-life processes, recruiters did not really “choose” to use ADSSs; rather, they were required to adopt the technology. Taking into account costs and implementation issues of ADSS solutions, using or not using these systems in an organization is a strategic decision made by top management, and recruiters must abide by this decision. In Ochmann et al.’s (2021) study, a mandatory context strengthened aversion for ADSSs in users’ discourses more than a voluntary context. However, Ochmann et al. did not measure actual behaviors but attitudes, which leaves an important avenue of research on the link between attitude and behavior in predictive hiring. Our own results suggest a lack of consistency between attitudes and behaviors in algorithm-assisted resume screening.

## Conclusion

Because of the scarcity of research in preselection and the high stakes of the preselection outcomes, we aimed to explore the mechanisms underlying recruiters’ behavior toward ADSS. In an experiment comparing ADSS and human expert respective influence on attitudes and choices of actual recruiters, we demonstrated the discrepancy between recruiters’ stated trust in a human expert and their reliance behavior towards an inconsistent algorithmic recommendation. Such finding raises questions regarding the cognitive process leading recruiters to rely on an algorithmic advice in a complex and subjective task like resume screening.

## Data Availability Statement

The raw data supporting the conclusions of this article are available at https://osf.io/t5wsq/.

## Ethics Statement

Ethical review and approval was not required for the study on human participants in accordance with the local legislation and institutional requirements. The patients/participants provided their written informed consent to participate in this study.

## Author Contributions

All authors listed have made a substantial, direct, and intellectual contribution to the work and approved it for publication.

## Funding

This research was financed by a French National Research Agency grant (ANR-15-IDEX-02) in collaboration with Grenoble Alpes University (IRGA).

## Conflict of Interest

The authors declare that the research was conducted in the absence of any commercial or financial relationships that could be construed as a potential conflict of interest.

## Publisher’s Note

All claims expressed in this article are solely those of the authors and do not necessarily represent those of their affiliated organizations, or those of the publisher, the editors and the reviewers. Any product that may be evaluated in this article, or claim that may be made by its manufacturer, is not guaranteed or endorsed by the publisher.
