# Supplementary material for: Should I Trust the Artificial Intelligence to Recruit? Recruiters’ Perceptions and Behavior When Faced With Algorithm-Based Recommendation Systems During Resume Screening
Source: Front Psychol. 2022 Jul 6;13:895997. doi: 10.3389/fpsyg.2022.895997 (PMC9298741; doi:10.3389/fpsyg.2022.895997)

APPENDIX

Table S1: Correlation matrix

Variable	Min	Max	M	SD	1	2	3	4	5	6	7	8	9	10	11	12	13	14	15
1. Diff_score	-4	4	0,40	1,40	—														
2. Trust in ADSS recommendation	2	9	6,56	1,31	0,01	—													
3. Trust in expert reco.	4	9	7,00	1,07	0,12*	na	—												
4. Propensity to trust automation	1,4	7	4,57	1,04	-0,02	0,61***	na	—											
5. Extraversion	1	7	4,66	1,33	0,05	0,16**	0,14*	0,13*	—										
6. Agreeableness	1	7	4,33	1,11	-0,01	0,11	0,15*	0,12*	0,21***	—									
7. Concientiousness	2	7	5,75	0,99	0,05	0,07	0,26***	0,06	0,17***	0,08*	—								
8. Neuroticism	1	7	3,32	1,32	-0,03	-0,08	-0,14*	-0,11	-0,21***	-0,38***	-0,24***	—							
9. Openess	1	7	4,70	1,23	-0,02	0,09	0,12	0,13*	0,18***	0,14***	0,10*	-0,13***	—						
10. Self competence	1	7	5,71	1,08	0,05	0,21***	0,28***	0,12	0,26***	0,10***	0,22***	-0,22***	0,18***	—					
11. Compare self with ADSS	3	9	6,04	1,36	-0,14*	0,50***	na	0,49***	0,05	0,02	0,09	-0,09	0,11	0,13*	—				
12. Compare self withexpert	2	9	6,13	1,37	0,11	na	0,40***	na	-0,01	-0,02	0,13*	0,00	0,04	0,07	na	—			
13. Recruiter expertise	0	3,4	1,67	0,81	-0,04	0,05	0,11	0,04	0,16***	0,04	0,07	-0,07	0,05	0,33***	-0,01	0,13*	—		
14. Age	20	69	40,60	10,06	-0,06	-0,01	0,03	0,07	0,14***	0,09	0,17***	-0,07	0,08*	0,20***	0,04	0,02	0,31***	—	
15. ADSS_use (1=no, 2=yes)					-0,01	0,00	0,03	0,13*	0,05	-0,02	-0,03	-0,03	0,00	0,05	-0,04	0,11	0,10*	-0,05	—
16. Gender (1=M, 2=F)					0,05	0,06	-0,05	0,03	-0,03	-0,03	0,09*	0,11**	0,00	-0,10**	-0,01	-0,10	-0,04	-0,10*	-0,01

* p < .05, ** p < .01, *** p < .001

Figure S1: Resumes scores distribution according to experimental conditions and resumes' suitability

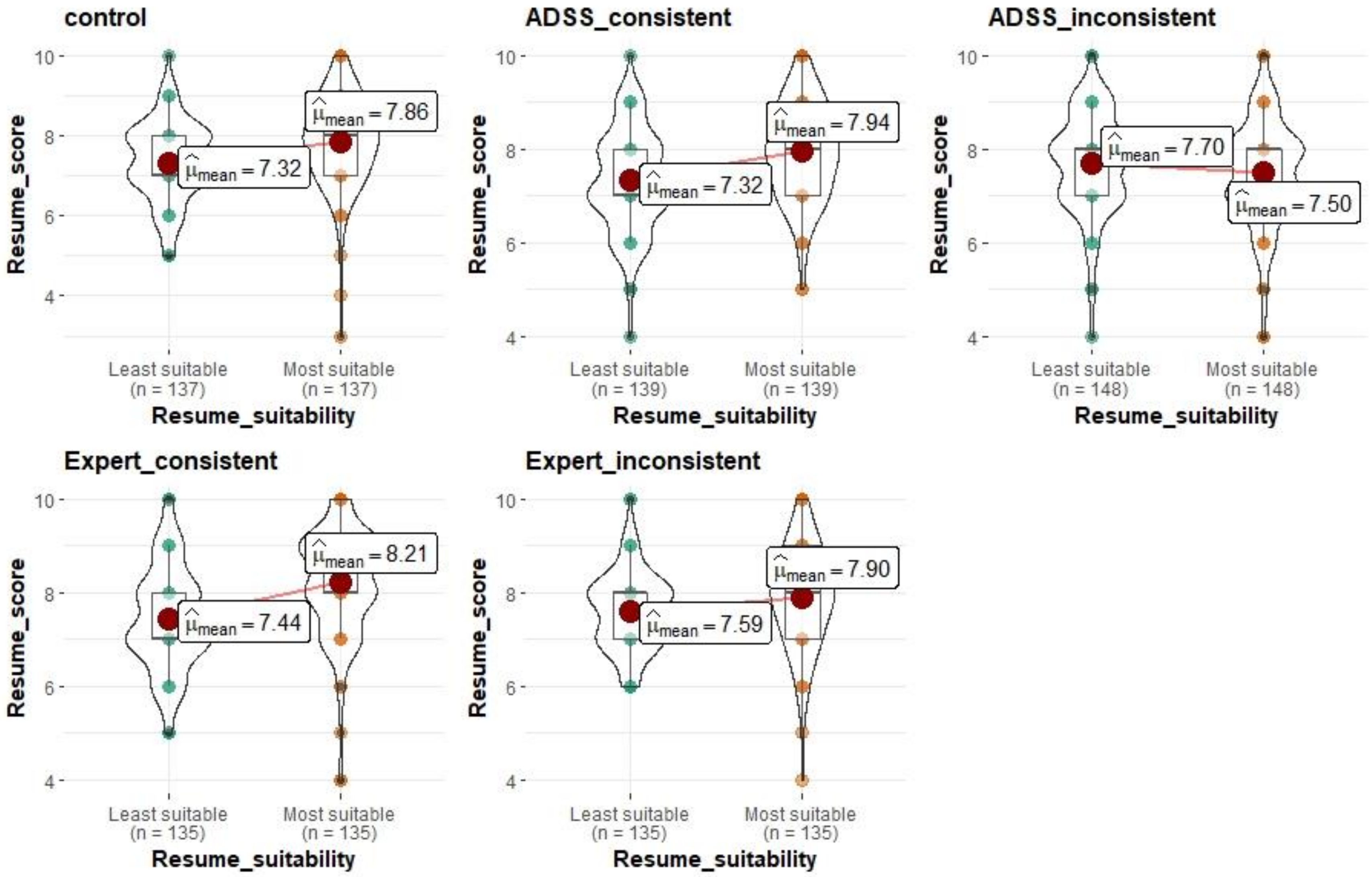

Figure S2: Distribution of scores of Post-Task Trust across experimental conditions

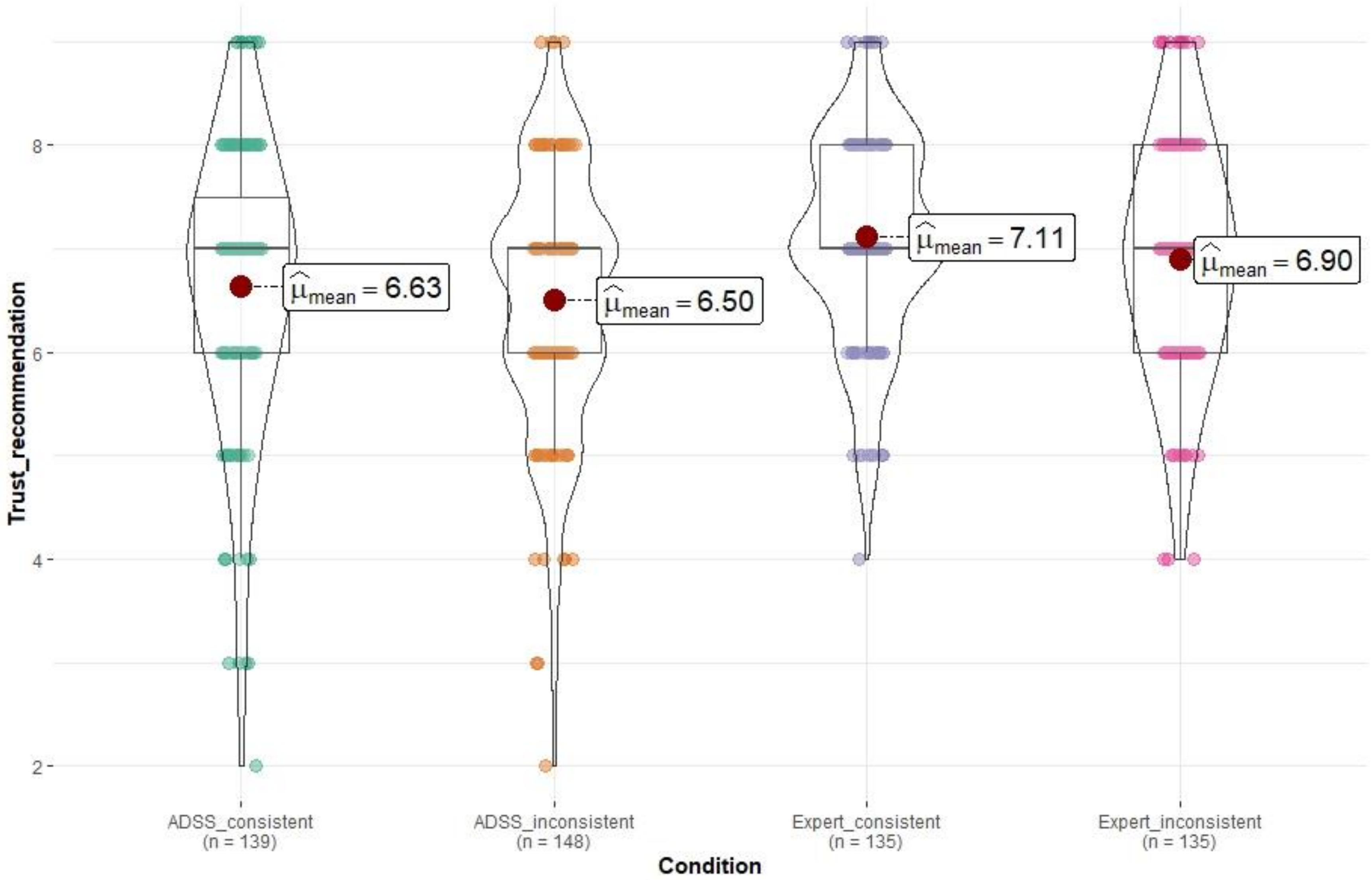

Figure S3: Distribution of diff-scores across experimental conditions

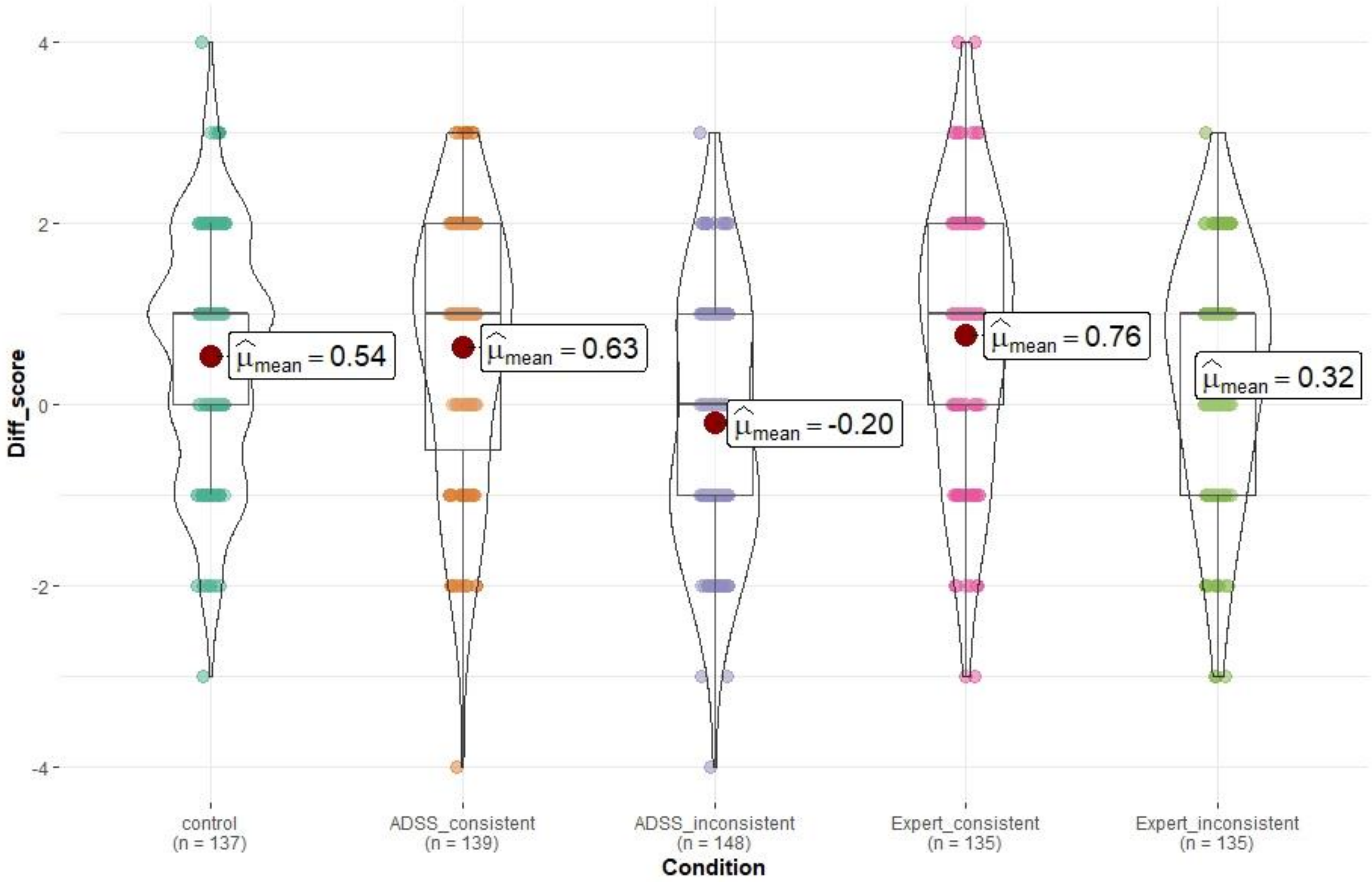

Figure S4: Types of resumes ranked in first position across experimental conditions

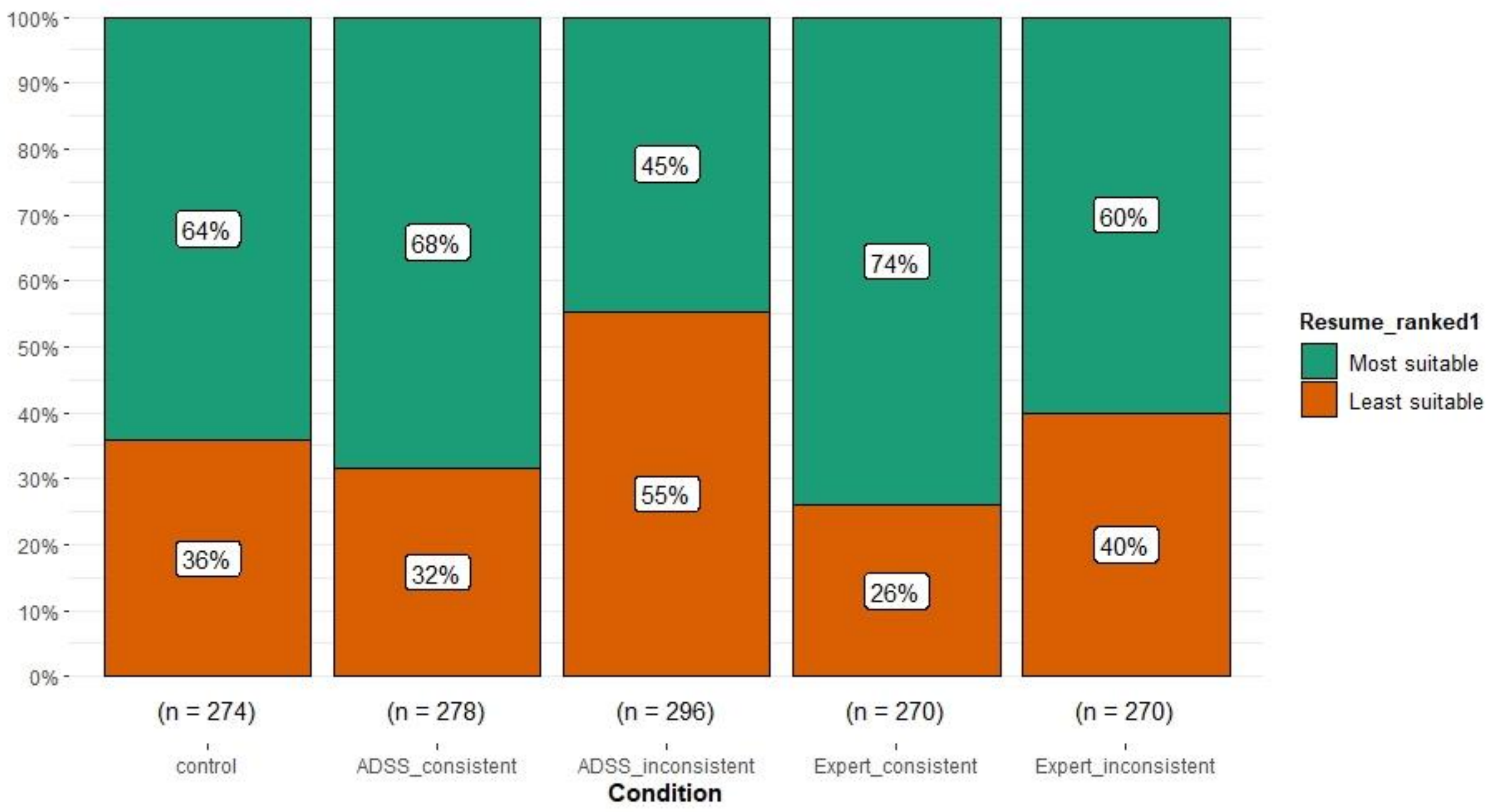

Supplement: Supplementary file 1 [file Data_Sheet_1.pdf]
